# Oligometastatic Prostate Cancer: Clues from an N-of-1

**DOI:** 10.3390/jcm15103910

**Published:** 2026-05-19

**Authors:** Alexander Kirschenbaum, Parisa Verma, Pamela Cheung, Shen Yao, Christopher Drummond, Isabella Tipi, Andy Yao, Alice C. Levine

**Affiliations:** 1 Department of Urology, Icahn School of Medicine at Mount Sinai, New York, NY 10029, USA; 2 Division of Endocrinology, Diabetes and Bone Diseases, Department of Medicine, Icahn School of Medicine at Mount Sinai, New York, NY 10029, USA; 3 Department of Oncological Sciences, Icahn School of Medicine at Mount Sinai, New York, NY 10029, USA

**Keywords:** prostate cancer, oligometastases, castration-resistance, PSMA, PAP, plasticity, hypoxia

## Abstract

**Background/Objectives**: Metastatic Prostate Cancer (mPCa) is generally treated with systemic therapy. Many of these treatments, particularly androgen ablation, are not curative and cause substantial morbidity. Oligometastasis is defined as a limited number of metastatic deposits, whose disease does not seem to progress to a widespread distribution of cancer. There have been some reports of trials of localized treatment of PCa oligometastatic disease with curative intent. We herein report a case of oligometastatic prostate cancer treated primarily with surgical removal of metastases, which has no evidence of active disease twenty years post-operatively. **Methods**: Extensive retrospective chart review, immunohistochemical staining, growth rate calculations, and imaging studies were performed to trace the progression of this patient’s disease course. **Results**: A detailed investigation of biochemical markers of recurrence revealed normal-low prostate specific antigen (PSA) despite advanced disease, early rather than metachronous dissemination of metastases to distant sites, and hypoxia-conditioned phenotypic plasticity and memory in disseminated tumor cells (DTCs). **Conclusions**: This rare outlier case of oligometastatic prostate adenocarcinoma challenges traditional linear models of metastatic progression and clinical reliance on PSA as a marker for PCa detection and treatment in advanced cases. By investigating key questions regarding the identity, timing, and trajectory of DTCs, we propose a biologically informed narrative of this patient’s disease progression and a reconsideration of metastases directed therapy (MDT) of oligometastases as primary therapy in select patients.

## 1. Introduction

Prostate cancer (PCa) is a leading cause of cancer-related morbidity and mortality among men worldwide, with both global incidence and mortality projected to continue rising in the coming decades [1,2,3]. In the United States, PCa is the most commonly diagnosed malignancy in men, accounting for approximately 30% of all new male cancer diagnoses in 2025 and remains the second leading cause of cancer-related death after lung cancer [3].

The most significant predictor of mortality in PCa is the presence of metastases. Hormone-naive metastatic PCa is biologically heterogeneous, and may manifest anywhere across a spectrum of disease burden, metastatic patterns, and therapeutic responsiveness. Current standard-of-care treatment for newly diagnosed metastatic disease generally includes androgen deprivation therapy (ADT) with treatment intensification using anti-androgen agents or chemotherapy. However, these strategies are associated with significant risks, including cardiovascular morbidity and negative impacts on quality of life.

Oligometastatic PCa, traditionally defined as the presence of three to five metastatic lesions identified on conventional imaging modalities, represents a distinct clinical subset of metastatic disease. However, this definition is rapidly evolving with the increasing use of prostate-specific membrane antigen (PSMA) PET/CT, which detects substantially lower-volume disease than prior imaging approaches. As a result, the biological natural history of true oligometastatic PCa remains incompletely understood, particularly because most patients undergo early local radiation and/or systemic treatment, limiting opportunities to observe untreated disease progression.

We herein describe a case of high-grade, high-stage PCa initially managed with radical prostatectomy, followed shortly thereafter by oligometastatic recurrence in the pleura and a lymph node. Both metastatic lesions were surgically resected, and the patient remains without evidence of active disease more than twenty years post-operatively. This exceptional responder case [4] gives us some clues regarding the heterogeneity of this disorder. 

## 2. Case Presentation

A 58-year-old man with urinary hesitancy presented to his urologist. Digital rectal exam revealed a palpable right prostatic nodule and PSA level was 2.32 ng/mL. The patient then underwent transrectal needle biopsy which demonstrated prostatic adenocarcinoma (PCa) Gleason score of 7 (4 + 3). At the time, CT and bone scan revealed no evidence of metastases. The patient underwent laparoscopic radical prostatectomy with resection of the right neurovascular bundle. Final pathology report disclosed Gleason score of 9 (5 + 4) stage pT3a. One-month post-operation, the patient’s PSA level remained detectable at 0.28 ng/mL. The patient was then administered external beam radiation therapy to the prostatic bed. One-year post-operation, and 9 months post-RT, the patient’s PSA had risen to 1.37 ng/mL. The patient was restaged. The bone scan was negative. However, a deoxyglucose FDG-PET/CT revealed lesions in the right parietal pleura and right pelvic iliac lymph node (Figure 1A and Figure 1B, respectively). Biopsy of the pleural lesion was negative for solid tumor markers including PSA, suggesting a metastatic adenocarcinoma of unknown origin. The pleural lesion was surgically excised followed by radiation therapy to the pleural bed. The surgical pathology described a 4 cm mass “lying over” the parietal pleura. The patient sought a second pathological assessment which demonstrated positive prostatic acid phosphatase (PAP) staining, confirming prostatic origin. Immediate post-operative PSA was 0.28 ng/mL and decreased further to 0.12 ng/mL one month later confirming that this was a prostate cancer pleural metastasis. Of note, even after removal of the pleural lesion, the PSA did not drop to undetectable levels suggesting that the continued source of PSA was the lymph node. Over the next six months, PSA levels continued to increase. After a discussion with the patient, he opted to begin intermittent anti-androgen monotherapy (Casodex 150 mg daily). On the anti-androgen therapy the PSA became, for the first time, undetectable. Repeat PET/CT scan 3 months after initiation of Casodex therapy demonstrated decreased activity in the lymph node and after one year of Casodex therapy the PET/CT scan was negative for any activity. The Casodex was discontinued and PSA rose shortly thereafter. A repeat PET/CT done 9 months after discontinuation of Casodex demonstrated increased activity in the lymph node and the anti-androgen was restarted. The PSA response to the second round of Casodex therapy was more short-lived (3 months) and repeat PET/CT done one year after restarting Casodex monotherapy redemonstrated lymph node uptake. At this point, the patient was considered to have castration-resistant disease on monotherapy and the Casodex was discontinued. He had received a total of 24 months of intermittent androgen ablation monotherapy at this point. The lymph node lesion was resected and identified as 5.5 × 4 × 3 cm in size and prostate in origin (PAP+++, PSA+). The patient’s post-operative PSA dropped to <0.008 ng/mL, remaining undetectable over the next twenty years without further treatment. Figure 2 demonstrates his clinical course over 60 months, including serum PSA values, imaging studies and clinical events.

## 3. Discussion

### 3.1. Detection of Oligometastases

We herein present a case of hormone-naive mPCa characterized by high Gleason grade and low PSA primary disease. The patient had two demonstrable metastases, one to the parietal pleura of the lung and one to the right pelvic iliac lymph node. Approximately 50% of patients initially diagnosed with localized prostate cancer will develop metastatic disease. The most common metastatic sites include bone (84%), lymph nodes (10.6%), liver (10.2%), and thorax (9.1%). Importantly, the site of metastasis carries prognostic implications, with pulmonary and lymph node involvement generally associated with a more indolent clinical course compared with bone or visceral metastatic disease.

The patient met the conventional definition of oligometastatic disease, typically defined as 3–5 metastases detected on standard imaging modalities. The classification of disease as oligometastatic, however, is fundamentally limited by the sensitivity of available diagnostic tools. The number of metastases identified on imaging may not represent the true extent of dissemination but rather the burden of disease that surpasses current detection thresholds. Conventional modalities (CT/MRI/bone scan) require a much larger tumor burden for detection, with PET/CT imaging detecting lower burdens. Newer imaging modalities, particularly PSMA-PET/CT, improve sensitivity for detecting small-volume metastases up to 98% and are independent of hormonal status [5].

Widely considered the most sensitive modality, current PSA testing has an ultrasensitive cutoff of 0.001 ng/mL. Importantly, the relationship between tumor volume and PSA levels depends on the biological activity of tumor cells, particularly their secretion of PSA. Therefore, the assumption that PSA testing can reliably detect disease at lower tumor burdens may not hold true in tumors with low androgen receptor (AR) expression or poor differentiation, where PSA production may be diminished despite significant tumor burden [6], as in our case.

In our patient we were able to roughly calculate the number of tumor cells removed in the pleural lesion by determination of the volume of the tumor removed (4 grams) combined with calculation of the percent of PCa cells on H&E sections (25%) (Appendix A). Thereafter, we divided the calculated number of cells in the pleural lesion by the change in PSA levels pre and post its removal. Table 1 gives an estimated correlation between PSA serum levels and tumor cell number in this patient. Of note, in our patient, who presented with low-PSA poorly differentiated prostate cancer, even at very low levels of PSA (<0.01 ng/mL), there can be 10 million tumor cells present.

Consequently, traditional surveillance methods may underestimate the true extent of disease. In our patient, following resection of the pleural metastasis, the PSA remained low but detectable at 0.12 ng/mL (Figure 2). At that time, the patient harbored a 4-cm lymph node metastasis, corresponding to an estimated tumor burden exceeding 2 billion cells. This observation raises the possibility of small, low-PSA-producing tumor cells that may have disseminated early from the primary in tumor development, seeding oligometastatic lesions well before clinical detectability. As a result, deciphering whether metastases arise early or late in disease progression (i.e., synchronous versus metachronous, or parallel versus linear evolution) remains fundamentally constrained by the sensitivity of current detection methods. This limitation raises a critical question in cancer biology: when do metastatic cells actually first depart from the primary tumor?

### 3.2. Timing of Metastases: Synchronous vs. Metachronous

The time to metastasis detection (fewer than eighteen months after treatment of the primary) suggests a model of early dissemination, consistent with the parallel progression hypothesis proposed by Klein [7]. In contrast to Leslie Fould’s widely accepted linear progression model, wherein metastases are only seeded once the primary tumor has undergone extensive evolution, the parallel progression model posits that metastasis is initiated long before clinical detection of the primary tumor [8]. This framework is supported by Klein’s proposed “lead-time effect,” whereby the combination of early dissemination and metastatic growth rates twice those of the primary may enable metastases to “catch up,” presenting clinically almost synchronously with a developed primary tumor.

Our patient’s prostate lesion was detected during a routine urologic examination, and FDG-PET/CT scans revealed pleural and lymph node metastases just over one year later (Figure 1). Metastases detectable by FDG-PET/CT one year after initial PCa diagnosis would be expected to have reached volumes on the order of 10 mm^3^ to 1 cm^3^, corresponding to approximately 10^7^ to 10^9^ tumor cells and requiring roughly 20 to 30 doublings from a single disseminated tumor cell (DTC). The established tumor volume doubling time (TVDT) for primary prostate tumors is approximately 150 days, while metastatic lesions exhibit a shorter TVDT of approximately 75 days [9] (Figure 3).

If we assume that metastatic growth initiated from a single DTC at the time of primary tumor detection, the lymph node and pleural lesions would have required a TVDT of approximately 16.7 days to reach radiographically detectable sizes within the observed eighteen-month timeframe. This rate represents only a fraction of the established metastatic doubling time of about 75 days and is therefore inconsistent with known prostate cancer kinetics. Taken together with his detectable PSA post-prostatectomy and the continuous rise in PSA in the 18 months post-surgery even after external beam radiotherapy, it seems highly likely that his oligometastases were present at the time of detection of his primary lesion.

These data suggest that oligometastatic dissemination occurred early in the course of this patient’s disease, consistent with the parallel progression model of metastasis. This observation raises a second critical question: which tumor cell populations disseminated at this early timepoint and gave rise to the metastatic lesions?

### 3.3. Clonal Origin of Metastases

Prior studies have shown that most prostate cancer metastases are monoclonal in origin, arising from a single dominant clone within the primary tumor [10,11,12,13]. To investigate the clonal origin of the oligometastatic lesions in this patient, we performed immunohistochemical and histopathological analyses of the primary and metastatic sites.

Our patient’s prostate tumor exhibited notable clonal heterogeneity, with two spatially and morphologically distinct epithelial clones identified within the primary lesion, represented by the two boxes in Figure 4. The peripherally located clones (Figure 4, Box A, clones 2, 3, 4, 5) were Gleason 9 (5 + 4) and demonstrated markedly reduced PSA expression, but retention of AR and PAP (Figure 5). In contrast, the centrally located tumor clone was Gleason 7 (3 + 4) (Figure 4, Box B, represented by clone 1) and exhibited high levels of PSA (PSA+++), while also remaining positive for AR and PAP (Figure 5).

Interestingly, even within the peripheral Gleason 9 clones, a spatial gradient of PSA expression was observed. Clones located closer to the central Gleason 7 tumor retained low levels of PSA expression, whereas the most peripheral clones (clones 4 and 5) were virtually PSA negative. These PSA-negative clones reside in less vascularized regions of the prostate, suggesting that their low-PSA phenotype may reflect adaptive responses to local oxygen availability. Given the low PSA staining in the metastases, an initial pathology report could not conclusively identify the lesions as prostate in origin. However, PAP was diffusely positive in all lesions, confirming prostatic origin even where PSA expression was weak (Figure 5D,H,L,P).

### 3.4. Phenotypic Plasticity and Memory

AR expression was more pronounced in the metastatic sites compared to the primary tumor (Figure 5C,G,K,O). This pattern is consistent with phenotypic plasticity, the ability of tumor cells to reversibly alter their phenotype in response to environmental conditions to enhance survival or fitness [14].

Closely related is the concept of phenotypic memory, whereby cells retain adaptive behaviors acquired in prior microenvironments even after those stimuli are no longer present [15]. Taken together, IHC suggests that the low-PSA, low-AR cribriform clones located at the periphery of the primary tumor (Figure 4, clones 2–5) gave rise to metastases. We postulate that these cells exhibited both phenotypic plasticity and memory in the metastatic sites, re-establishing AR expression and signaling (i.e., PSA expression) in the relatively well-oxygenated lymphatic channels of the cortex (Figure 5N). The PCa expression profile of DTCs in the cortex resemble Clone 1 in the primary, an area that was also relatively well oxygenated.

### 3.5. Hypoxia as a Driver of Tumor Plasticity

Hypoxia is a major driver of this phenotypic plasticity. It is well known that hypoxia is a morphogen that regulates progenitor cell differentiation and cell fate decisions. In cancer, this mechanism is co-opted such that tumor-initiating (TICs) and disseminated tumor cells (DTCs) are often maintained within hypoxic niches in both primary and metastatic sites [16].

At the molecular level, hypoxia promotes such cellular adaptation through stabilization of hypoxia-indicible factors (HIFs), transcriptional regulators that activate genes required for survival under low oxygen conditions [16,17]. One important downstream effector of HIF signaling is MUC1-C, a well-characterized oncoprotein implicated in tumor progression and metastatic survival across cancers [18,19,20,21,22,23]. One particular feature of this patient’s low-PSA peripheral clones in the primary (Figure 4, clones 4 and 5) was localized MUC1-C positivity along their outer edge (Figure 6A). Immunohistochemical analysis confirmed that MUC1-C expression was preserved in the hypoxic areas of the metastatic sites (Figure 6F,K). Double staining for MUC1-C and AR (Figure 6B,G,L) and MUC1-C and Ki67 (Figure 6C,H,M) demonstrated that MUC1-positive cells (brown) were AR-negative and Ki67-negative, consistent with the idea that tumor initiating cells (TICs) residing in hypoxic niches in the primary and metastatic sites are undifferentiated and quiescent [24,25,26,27]. Of note, both PSMA and PAP are expressed in the primary, pleural and lymph node metastatic sites, but PAP (Figure 6O) is more uniformly expressed than PSMA (Figure 6N) in the hypoxic lymph node hilum.

At the same time, the metastatic lesions displayed robust AR expression (Figure 6G,L: red) and distinct Ki67-positive populations (Figure 6H,M: red) outside of MUC1-positive regions, indicating that tumor cells retain the capacity to transition between quiescent progenitor-like states and proliferative differentiated states depending on the oxygen gradient. 

These findings suggest that the initial clone that gave way to metastases capable of such dynamic phenotypic adaptation likely emerged from the hypoxic, MUC-1 positive edge of the primary tumor. Indeed, it has been reported that MUC1-C regulates lineage plasticity in prostate cancer. However, this interpretation of phenotypic plasticity warrants caution as we cannot confirm a causal relationship between hypoxia, MUC1-C expression and metastatic behavior based solely on immunohistochemistry.

The next question concerns the route these hypoxia-adapted DTCs took to reach their metastatic sites. Guided by the observation that in our patient, DTCs appeared to preferentially localize to hypoxic niches—such as the lymph node hilum—we next examined the morphology and immunoprofile of tumor clusters within periprostatic lymphatic channels and the right pelvic iliac lymph node in order to reconstruct a potential route of metastatic spread.

### 3.6. Route of Tumor Dissemination

Histologic examination revealed strong evidence that lymphatic rather than hematogenous dissemination accounted for the lymph node metastasis (Figure 7). Tumor cells were not identified in the vasculature of the primary or metastatic lesions. Tumor cell clusters identified within periprostatic lymphatic channels stained diffusely positive for PAP (Figure 7A). PSA and AR also showed the same staining pattern. We labelled the afferent lymphatics in the cortex of the lymph node with D2-40 (Figure 7C). DTCs were found within the afferent lymphatics, which maintained PSA expression once inside the lymphatic channels, which is a better oxygenated environment (Figure 7B,D).

### 3.7. Fate of DTCs Within the Lymph Node

We established that DTCs arrived in the lymph node through the afferent lymphatics in the subcapsular region, however, IHC demonstrated that the entire lymph node was replaced by tumor with only a rim of compressed germinal centers near the lymph node capsule remaining (Figure 5M,N,O,P). This intranodal displacement of germinal centers to the periphery, suggested that DTCs in the afferent lymphatics (Figure 7D) travelled via the medullary sinus to the hypoxic hilum. Figure 8A demonstrates HIF1alpha expression in the lymph node metastasis and confirms the hypoxic gradient with lowest O_2_ concentration and highest HIF1 alpha expression in the hilum. The overlaid triangle depicts the generalized oxygen tension (pO_2_) gradient from the well-oxygenated capsule and subcapsular regions toward progressively lower oxygen levels at the hilum, consistent with the observed spatial pattern of staining. Figure 8B is the proposed theoretical route of DTCs within the lymph node, entering via the afferent lymphatics eventually reaching hilum via the subcortical and medullary sinuses. We speculate that they differentiate as they proliferate centripetally toward the more well oxygenated regions of the lymph node, compressing the germinal centers. It is important to note that prior to the lymph node resection, the patient received 24 months of intermittent androgen ablation monotherapy with Casodex, a known activator of hypoxic signalling via HIF1a [28]. This increased hypoxia may have expanded the progenitor cell population in the lymph node as compared to the hormone naive prostate and pleural lesions.

### 3.8. Clonal Origin and Route of Dissemination of Pleural Metastasis

Examination of pleural mets did not disclose any evidence of DTCs in the vasculature but did show some DTCs in lymphatics. We speculate that DTCs in the lymph node hilum disseminated via the efferent lymphatics to the region of the parietal pleura (Figure 9B). Figure 9A is a proposed overall schematic of the route of the DTCs from the prostate to the lymph node and then to the outer pleural region based on our immunohistochemical analysis. In all three sites (prostate, lymph node, and pleura), PCa cells demonstrated plasticity with dedifferentiation in the most hypoxic areas (red) and redifferentiation as oxygen levels rise in blue and green areas.

## 4. Conclusions

We herein present a case of PCa with no evidence of disease twenty years after removal of pleural and lymph node oligometastases. The patient’s initial presentation (low PSA, high Gleason Grade, high clinical stage PT3a) all portended a poor prognosis. One year after radical prostatectomy, no metastases were detectable by bone scan and traditional CT. However, his PSA never normalized after radical prostatectomy and external beam radiation therapy indicating that he had oligometastases that were synchronous with his primary tumor presentation but not demonstrable with the radiographic tools available at the time. Because his PSA continued to rise, he underwent deoxyglucose PET/CT that demonstrated oligometastases in the pleura and the lymph node.

Oligometastatic hormone-sensitive prostate cancer is a heterogeneous disease. This entity poses a significant challenge for clinicians, as controversies exist regarding the definition, diagnostic significance and treatment of patients with a limited number of metastases. In the past using techniques such as conventional CT and bone scan, many oligometastases were missed until they were quite large, introducing lead time bias. In those older studies, patients with presumed metachronous disease had better outcomes than those with synchronous oligometastases. Newer, more sensitive imaging techniques such as PSMA-PET/CT are challenging our concept of synchronous vs. metachronous metastases, as oligometastases may be picked up much earlier. In our patient, by studying his case history combined with calculations derived from his serum PSA vs.the volume of his surgically-removed metastases, we estimate that both metastases were present at prior to the detection of the primary, implying that his metastases were synchronous rather than metachronous. In spite of early dissemination there appears to have been limited metastatic potential and he had durable remission after metastases-directed therapy (MTD).

In this case, the tumor initiating cells (TICs), disseminated tumor cells (DTCs), and metastatic initiating cells (MICs) exhibited the same programmed plasticity based on oxygen gradients in primary and metastatic sites. This patient’s TICs, DTCs, and MICs demonstrated preservation of the hierarchical differentiation observed in the primary tumor, even as they became castration-resistant in the lymph node after Casodex monotherapy. We speculate that these features of his tumor cells, epigenetic plasticity and memory of the normal differentiation program, contributed to the limited metastatic potential in this case.

The immunohistochemical characteristics, timing/route of dissemination, tumor growth rates, and plasticity of PCa in this patient could only be studied in retrospect with removal of oligometastases. In addition, findings from this unique prostate cancer are not generalizable to all patients with PCa oligometastases. However, this case informs us that definitions of PCa oligometastases, low metastatic burden and synchronous vs. metachronous spread are fluid and dependent on the sensitivity of the available detection methods. It also underscores that not all oligometastases will progress even with unfavorable prognostic markers. Clearly our patient’s prostate cancer was lacking many of the features needed for a successful metastatic cascade. The most important clue was that there was no progression of early oligo-metastastases for some time prior to removal of both the pleural and lymph node metastases.

If this patient presented today, he would have had PSMA-PET CT that likely would have detected synchronous oligometastases at the time of presentation given the PSMA positivity on IHC of the primary and metastases. A recent meta-analysis that included six randomized trial comparing MDT plus standard of care (SOC) (generally anti-androgen therapy) vs. SOC alone showed that the addition of MDT improved progression-free survival, radiological progression-free survival and castration resistance-free survival [28,29]. There are few reported studies utilizing MDT alone and even fewer that include surgical resection of metastases as MDT vs. observation [29,30]. The 2018 STOMP trial results showed that MDT—using surgery or stereotactic body radiotherapy (SBRT)—significantly delays the need for androgen-deprivation therapy (ADT) in oligometastatic prostate cancer patients [30,31]. There are at least 10 ongoing randomized trials of oligometastasis local therapy but nine of ten compare MDT plus ADT to ADT alone and the remaining trial (ADOPT-NCT04302454) compares MDT with radiation to MDT radiation plus ADT [31,32]. While these ongoing trials may yield important data about the value of MDT in select patients with oligometastases they may not lead to ADT-sparing regimens for this population. The current AUA guidelines for treatment of synchronous/de novo low volume metastatic hormone-sensitive prostate cancer recommend intensification with androgen deprivation therapy (ADT) plus novel hormonal agents or docetaxel and consideration of radiation to the oligometastases. Although this patient had some transient anti-androgen therapy, he was spared twenty years of systemic therapy with all the associated side effects and comorbidities. In addition, during these past twenty years he had significant cardiac disease necessitating coronary artery bypass surgery and pacemaker placement even without ADT. In the era of sensitive imaging techniques there will be more oligometastases detected and clearly additional biomarkers are needed to guide the timing and extent of systemic therapy in these cases. This compelling N-of-1 prostate cancer case highlights the heterogeneity of oligometastatic disease, the challenges in identifying the most effective personalized treatment and the need for more studies to evaluate MDT alone in selective patients with more indolent disease.

## Figures and Tables

**Figure 1:**
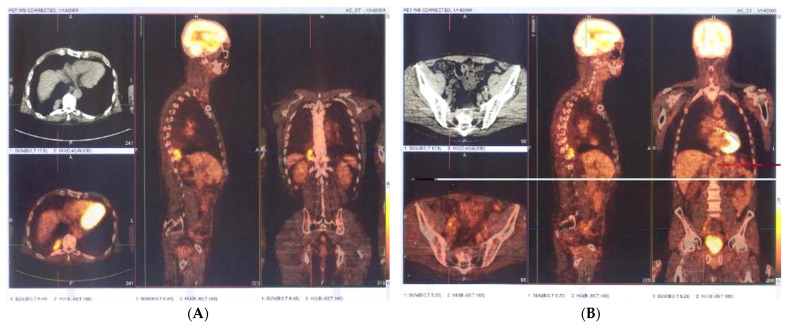
FDG-PET/CT 13 months post radical prostatectomy. (**A**) Arrow indicating the location of the right pleural lesion, (**B**) right pelvic lymph node.

**Figure 2:**
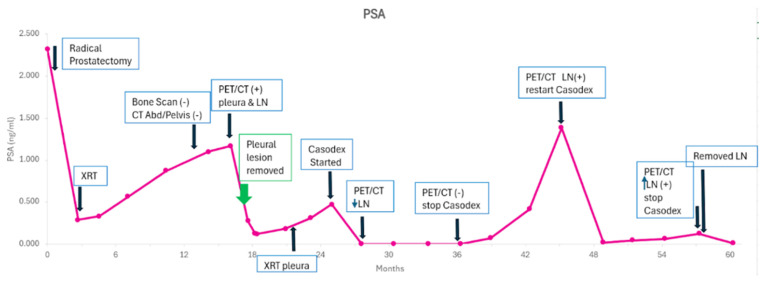
Serum PSA (ng/mL) plotted against months from initial diagnosis, along with significant imaging studies and clinical events.

**Figure 3:**
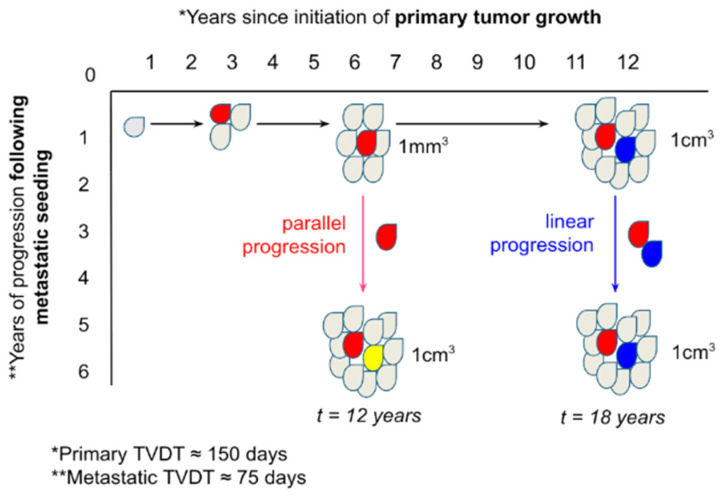
Conceptual model of linear versus parallel metastatic progression during prostate cancer evolution. The graph illustrates two proposed routes of metastatic spread as a function of time from initiation of primary tumor growth (top x-axis) and years following metastatic seeding (y-axis). Assuming a primary tumor volume doubling time (TVDT) of approximately 150 days, the primary lesion reaches ~1 mm^3^ by year 6 and ~1 cm^3^ by year 12. Metastatic lesions are modeled with a faster TVDT of approximately 75 days. In the parallel progression model (red arrow), dissemination occurs early when the primary tumor is still small, allowing the metastatic clone to evolve independently at the distant site (yellow clone), reaching ~1 cm^3^ at t = 12 years. In the linear progression model (blue), dissemination occurs later from a more evolved ~1 cm^3^ primary tumor, resulting in a metastasis that closely mirrors the dominant advanced primary clone and becomes detectable only at t = 18 years.

**Figure 4:**
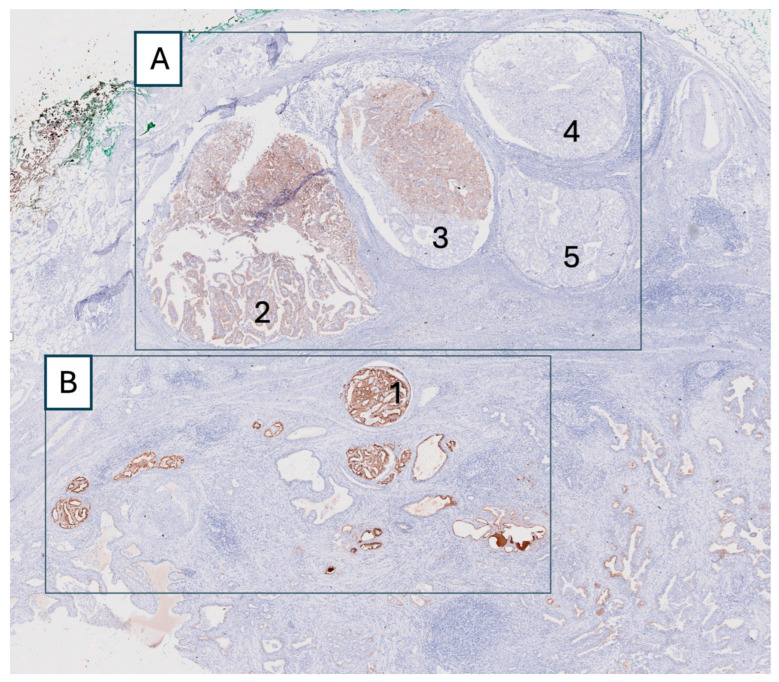
Radical prostatectomy specimen stained for PSA demonstrating several morphologically distinct clusters. Box A represents four clones (2–5) of Gleason score 9 (5 + 4) located peripherally with varying PSA staining from low to none. Box B represents Gleason score 7 (3 + 4) located centrally demonstrating strong and uniform PSA staining (Clone 1).

**Figure 5:**
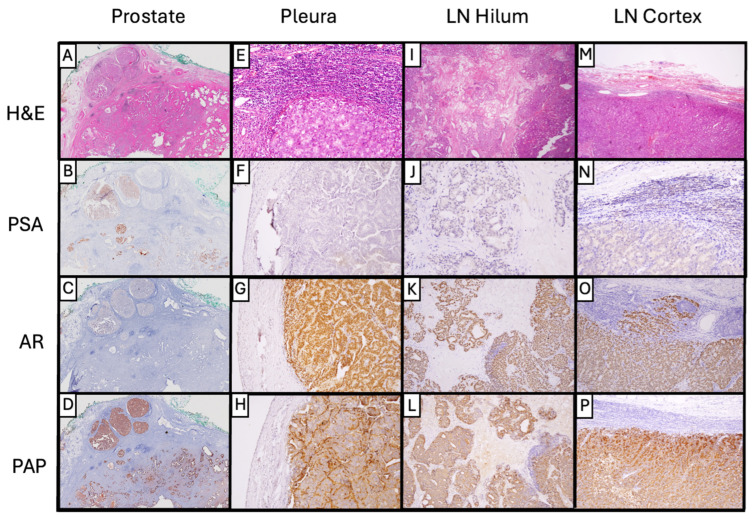
Comparison of PSA, AR and PAP expression in primary tumor, Lymph node (LN) cortex, LN-hilum and pleural metastases. H&E of primary (**A**) pleura (**E**), lymph node hilum (**I**) and lymph node cortex (**M**). PSA staining of primary (**B**) pleura (**F**), lymph node hilum (**J**), and lymph node cortex (**N**). AR staining of primary (**C**), pleura (**G**), lymph node hilum (**K**), and lymph node cortex (**O**). PAP staining of primary (**D**), pleura (**H**), lymph node hilum (**L**), and lymph node cortex (**P**).

**Figure 6:**
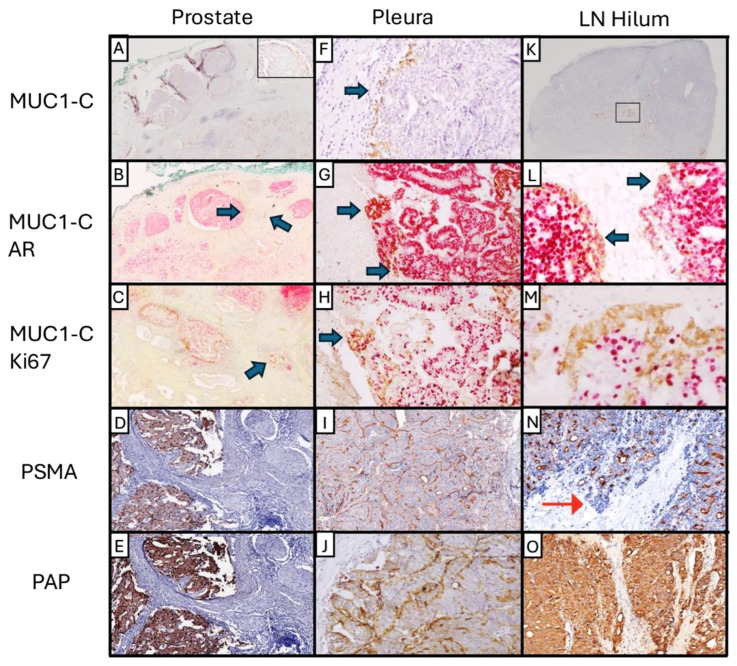
Immunohistochemical Expression of MUC1-C (brown), AR (red), Ki67 (red), PSMA (brown), and PAP (brown) in the primary prostate tumor (Panels (**A**–**E**)), the pleural metastasis (Panels (**F**–**J**)), and the lymph node metastasis in the hypoxic hilum (Panels (**K**–**O**)). Box in panel (**A**) indicates tumor initiating cells at the periphery of the clone (TICs). Arrows in panels (**B**,**C**,**F**,**G**,**H**,**L**) indicate MUC1-positive TICs. Box in panel (**K**) depicts the hilar region, expanded in panels (**L**–**O**).

**Figure 7:**
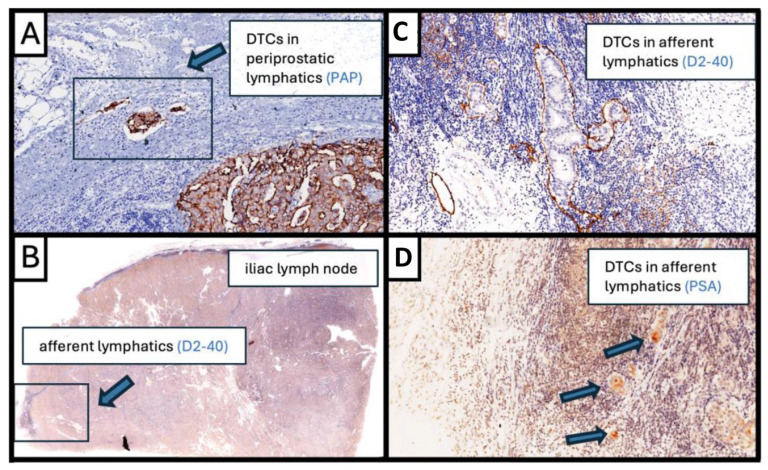
Histological Examination of Afferent Lymphatics: IHC for (**A**) PAP in periprostatic lymphatics, (**B**) PSA in lymph node metastasis, (**C**) D2-40 in afferent lymphatics, and (**D**) PSA in afferent lymphatics (high magnification) demonstrating that PCa cells leave the prostate via lymphatics and enter the LN capsule through afferent lymphatics. Arrows in Figure 7 are highlighting the findings in the text box of each panel.

**Figure 8:**
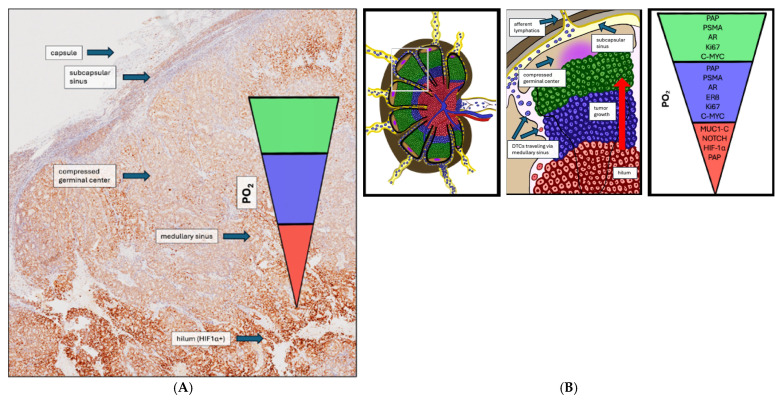
(**A**): Section of an iliac lymph node stained for HIF-1α, with the capsule, subcapsular sinus, compressed germinal center, medullary sinus, and hilar region labeled. (**B**): Proposed intranodal route of disseminated tumor cell progression along a hypoxic gradient. The left panel illustrates overall iliac lymph node architecture. The center panel provides a magnified schematic of the proposed metastatic route, in which disseminated tumor cells (DTCs) enter through the afferent lymphatics and subcapsular sinus, migrate through the medullary sinuses toward the hilum, and then compress germinal centers. Oxygen gradient: green highest, blue intermediate, red lowest.

**Figure 9:**
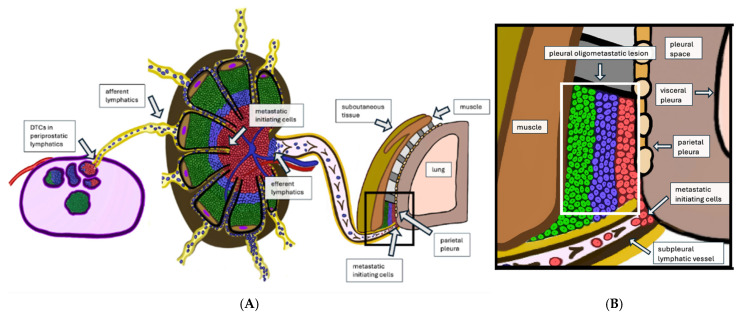
(**A**): Integrated model of lymphatic dissemination and oxygen gradient-driven phenotypic plasticity from primary to lymph node to pleural metastasis. (**B**): Proposed oxygen gradient-driven spatial phenotypic organization in the pleural oligometastatic lesion. The metastatic initiating cells are depicted entering through the subpleural lymphatic vessel, and the lesion is shown expanding outward from the parietal pleura, with spatially distinct cellular compartments corresponding to local oxygen availability and differentiation state. Oxygen gradient: green highest, blue intermediate, red lowest.

**Table 1:** Calculated correlation between serum PSA levels and tumor cell number in this patient.

PSA Level (ng/mL)	Calculated Cell Number Required for Detection in Our Patient
1.00	10^9^
0.10	10^8^
0.01	10^7^

## Data Availability

The original contributions presented in this study are included in the article. Further inquiries can be directed to the corresponding author.

## Abbreviations

The following abbreviations are used in this manuscript:

DTCs	disseminated tumor cells
mPCa	metastatic prostate cancer
MICs	metastasis initiating cells
PCa	prostate cancer
PAP	prostatic acid phosphatase
PSA	prostate specific antigen
PSMA	prostate specific membrane antigen

## Appendix A

Calculation for the correlation between serum PSA levels and tumor cell number in this patient, based on the patient’s PSA levels pre- and post-removal of the pleural lesion, and volume of tumor removed.
